# The Chinese version of the Health Professional Communication Skills Scale: Psychometric evaluation

**DOI:** 10.3389/fpsyg.2023.1125404

**Published:** 2023-08-09

**Authors:** Xiaoying Zhong, Fangmei Tang, Dongmei Lai, Xiujing Guo, Xiaorong Yang, Rong Hu, Dehua Li, Yongguang Lu, Sixu Liu, César Leal-Costa

**Affiliations:** ^1^Department of Nursing, West China Second University Hospital, Sichuan University, Chengdu, Sichuan, China; ^2^Laboratory of Birth Defects and Related Diseases of Women and Children (Sichuan University), Ministry of Education, Chengdu, Sichuan, China; ^3^Department of Child Rehabilitation, Chengdu Integrated TCM Western Medicine Hospital, Chengdu First People's Hospital, Chengdu, Sichuan, China; ^4^Chengdu Women's and Children's Central Hospital, School of Medicine, University of Electronic Science and Technology, Chengdu, Sichuan, China; ^5^Mianyang Central Hospital, University of Electronic Science and Technology, Mianyang, Sichuan, China; ^6^Faculty of Nursing, University of Murcia (UM), Murcia, Spain

**Keywords:** health professional, communication skills, reliability, validity, psychometric properties

## Abstract

**Objective:**

This study aims to translate the Health Professional Communication Skills Scale (HP-CSS) into Chinese and assess its psychometric properties.

**Methods:**

A total of 836 healthcare professionals were recruited. The demographic characteristics form and HP-CSS were used for data collection. The psychometric properties of HP-CSS were evaluated by examining item analysis, construct validity, known-group discriminant validity, internal consistency, and split-half reliability.

**Results:**

In terms of item analysis, the critical ratio (CR) of 18 items was both >3 (CR ranging from 9.937 to 28.816), and the score of each item was positively correlated with the total score (*r* ranging from 0.357 to 0.778, *P* < 0.001). The fit indices showed that the original correlated four-factor model of HP-CSS was adequate: χ^2^ =722.801; df = 126; χ^2^/df = 5.737; RMSEA = 0.075; CFI = 0.923; NNFI = 0.908; TLI = 0.906; IFI = 0.923. In terms of known-group discriminant validity, the HP-CSS total score was related to gender, occupation, work years, and communication skill training. Cronbach's α coefficient was 0.922, and the split-half reliability was 0.865 for the total scale.

**Conclusion:**

The Chinese version of the HP-CSS is a reliable and valid instrument to evaluate communication skills among healthcare professionals in China.

## Introduction

The relationship between healthcare professionals and patients has undergone significant changes since the second half of the 20th century, resulting in a more patient-centered model (Roter, 2004; Du et al., 2022). Communication can be regarded as the foundation for building interactions and relationships, and healthcare professionals' communication skills may refer to the provider's ability to convey knowledge, explanations, or instructions to the patient (Humphris, 2015; Bry et al., 2016). Patient-centered communication requires healthcare professionals to prioritize patient preferences, needs, and values (Saha et al., 2008; Maatouk-Buermann et al., 2016). China's National Health Plan for the 14th Five-Year Plan has pointed out that enhancing the medical service model and quality management is an essential element for comprehensively promoting the building of a healthy China (Poo, 2021). In fact, adequate communication skills are also recognized as one of the key clinical competencies for healthcare professionals (Rubinelli et al., 2019).

There is ample evidence showing that patient-centered communication among healthcare professionals, patients, and caregivers is integral to boosting patient satisfaction and treatment compliance, ultimately achieving optimal several health outcomes (Rock, 2021; Wolderslund et al., 2021). In terms of chronic care management, for example, diabetes and hypertension, effective health education will contribute to improving knowledge and understanding of illness and its probable consequences and adopting a healthier lifestyle (Claramita et al., 2020; Lambert et al., 2021). In terms of cancer care, good communication strategies will contribute to increasing screening and referral for anxiety and depression in patients with tumors (Moore et al., 2018; Shaw et al., 2022). Conversely, according to a report by the Chinese Pharmacists Association, 80% of patient complaints and medical disputes in the healthcare system have been linked to ineffective communication (Zhang and Sleeboom-Faulkner, 2011; Guo and Wang, 2021). Therefore, evaluating and training communication skills has become a high priority in treatment for all healthcare professionals (Wuensch et al., 2013; Humphris, 2015).

In China, few instruments are available for evaluating communication skills, including the Communication Skills Attitude Scale, the SEGUE Framework, and multisource feedback (Zhao et al., 2013; Zhang et al., 2018; Xiong et al., 2019). To the best of our knowledge, however, those tools are widely used among specific professional groups, such as medical students, trainee doctors, nursing probationers, physicians, and nurses (Mendi et al., 2020). Identifying a widely available, valid, and appropriate tool to assess healthcare professional communication skills may help advance the quality of care (Cubaka et al., 2018). The Health Professional Communication Skills Scale (HP-CSS) is a self-reported tool for testing communication skills in all healthcare professionals; some studies conducted in Spain, Turkey, and Iran have revealed that the HP-CSS has good psychometric properties (Leal-Costa et al., 2016; Julia-Sanchis et al., 2020; Nia et al., 2022).

Since the reliability and validity of HP-CSS have not yet been studied in China, the purpose of this study is to translate HP-CSS into Chinese and investigate its psychometric properties.

## Materials and methods

### Study design

**Figure 1:**
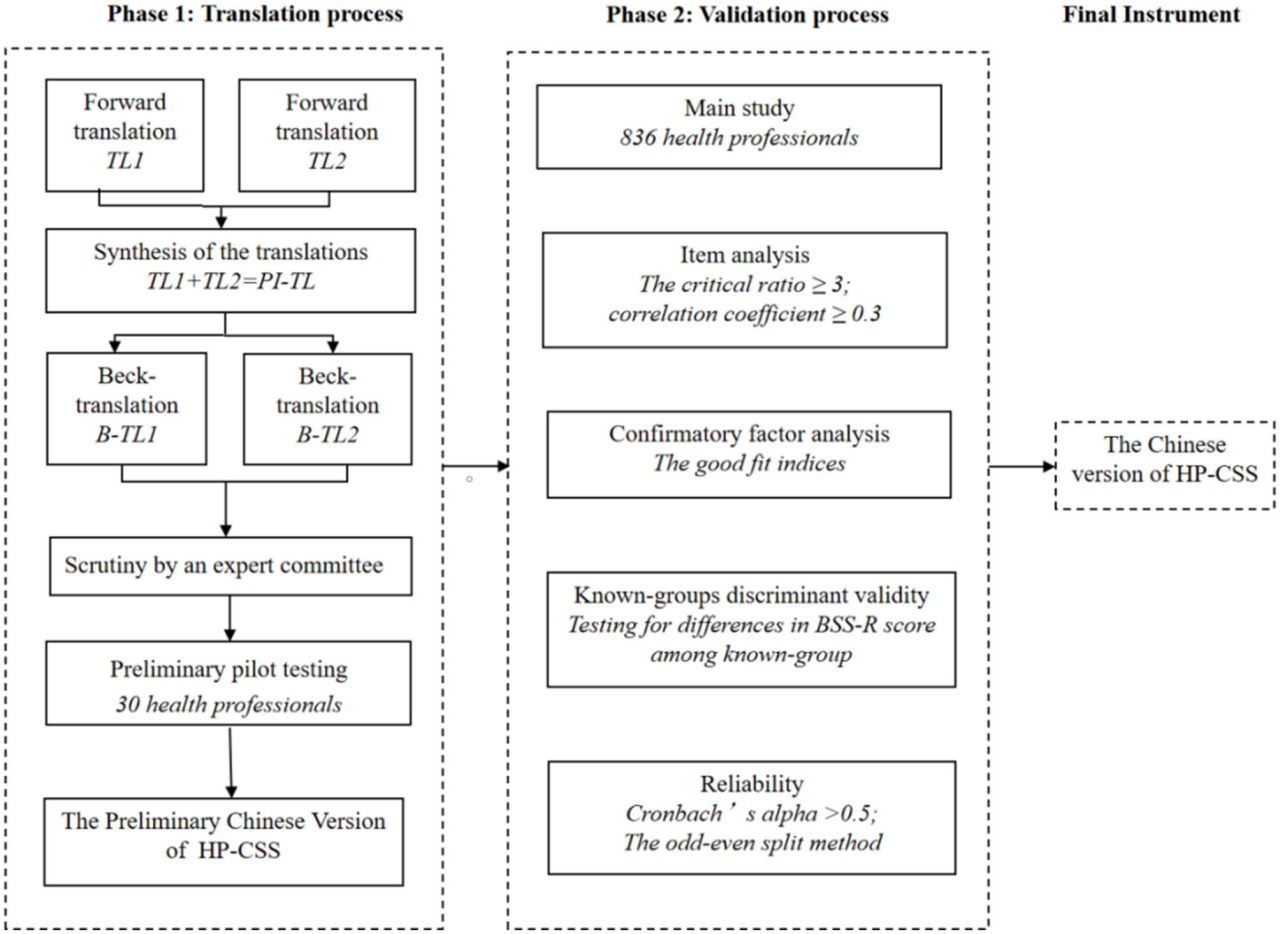
Translation and validation process of the HP-CSS.

This is a descriptive, cross-sectional, and methodological study. This study is divided into two phases. In phase 1, the HP-CSS was translated to Chinese following four steps: forward translation, back translation, scrutiny by an expert committee, and a pilot study. In phase 2, the psychometric properties of the Chinese version of the HP-CSS were verified through a cross-sectional survey (see Figure 1).

### Translation procedure

The permission for the translation and validation of HP-CSS was obtained from César Leal-Costa, the original developer of the scale. The following is the Beaton cross-cultural adaptation process (Beaton et al., 2000).

- *Step 1: Forward translation*. HP-CSS was independently translated into Chinese by two bilingual experts (a professor of public health and a doctor of evidence-based medicine) who were proficient in both English and native Chinese, forming T1 and T2. A panel including a nurse professor and three postgraduates in nursing reviewed the forward-translated versions to achieve the most accurate translation. After resolving ambiguities and disagreements, a preliminary initial translated version named version 1 was created (PL-TI).
- *Step 2: Backward translation*. An English teacher and a doctor of nursing were involved in this step, neither of whom had been exposed to the original HP-CSS before. Two researchers translated version 1 into English (B-TL1 and B-TL2) and compared it with the original scale.
- *Step 3: Scrutiny by an expert committee*. An expert committee of five was formed to evaluate cultural adaptability. The expert committee was made up of two associate professors of medical ethics, a professor of moral philosophy, and two professors of nursing, and the research directions are nursing management and nursing education, respectively.
- *Step 4: Preliminary pilot testing*. Convenience sampling selected 30 healthcare professionals for a preliminary survey and asked whether they had an unclear understanding of the content. The results showed that the healthcare professional had no unclear or ambiguous understanding of the items.

### Measures

#### Demographic characteristics form

Basic demographic information included age, education level, marital status, occupation, professional title, work years as a healthcare professional, and so on.

#### Health Professional Communication Skills Scale

The HP-CSS is a self-administered, multidimensional scale for evaluating the communication skills of healthcare professionals. It comprises 18 items classified into four domains: empathy (five items), informative communication (six items), respect (three items), and social skills (four items). Each item was scored on a six-point Likert-type scale from 1 to 6 (1 = almost never, 2 = once in a while, 3 = sometimes, 4 = normally, 5 = very often, and 6 = many times), except items 16 and 18, which are reverse scored. The internal consistency reliability of the HP-CSS was reported as 0.77, 0.78, 0.74, and 0.65 for empathy, informative communication, respect, and social skill, respectively (Leal-Costa et al., 2016).

### Participants

Participants were recruited using convenience sampling. The following were the inclusion criteria: (a) being aged 18–65 years; (b) being a healthcare professional in service; and (c) being able to read and write in Chinese. The healthcare professionals on probation and in practice were excluded.

### Sample size

The sample size was calculated according to the criteria required for factorial analysis; a sample of at least 200 participants was considered adequate (Marsh et al., 2014). A total of 982 healthcare professionals agreed to participate in the study; 146 individuals were excluded because they provided unreliable data. Finally, a total of 836 healthcare professionals were included in the data analysis.

### Setting and data collection

This study was conducted between March 2022 and September 2022. A Chinese free web-based platform (Sojump) was used for developing online questionnaires. We first sent the survey link to the hospital administrators in three tertiary hospitals in Chengdu, Sichuan Province, *via* communicative media (WeChat and QQ) and asked them to share it with healthcare professionals within reach. At the beginning of the online survey, informed consent was obtained from the participants. The privacy and anonymity of the healthcare professionals in the survey were assured.

### Data analysis

All data analyses were carried out using IBM SPSS Statistics for Windows, Version 21.0, and IBM AMOS Statistics for Windows, Version 24.0. All statistical tests were two-tailed, and a *P*-value of <0.05 was considered statistically significant.

### Demographic characteristics

For demographic characteristics, frequency and percentage were used to describe categorical and qualitative variables, while mean and standard deviation (*SD*) were used to show continuous variables with a normal distribution.

### Item analysis

Critical ratio and correlation coefficient methods were used for item analysis. First, the item scores of HP-CSS were summed up and then arranged in ascending order from high to low. The bottom 27% of the score was classified as the low score group (251 cases) and the top 27% as the high score group (244 cases), and the independent sample *t*-test was used to compare the two groups. The Spearman correlation coefficient was calculated between the item and the total score. In general, an item for which the absolute value of the critical ratio is <3 or an item for which the total correlation coefficient is <0.3 should be deleted (Livingston, 2011).

### Confirmatory factor analysis

The confirmatory factor analysis was used to evaluate the construct validity. When the Kaiser–Meyer–Olkin (KMO) test value was >0.6 and the Bartlett spherical test statistic was significant (*P* < 0.001), indicating the data was suitable for factor analysis (Geldhof et al., 2014). The evaluation indices of confirmatory factor analysis are relative chi-square (χ^2^/df), root mean square error of approximation (RMSEA), comparative fit index (CFI), non-normed fit index (NNFI), Tucker-Lewis index (TLI), and incremental fit index (IFI). A χ^2^/df ratio <6 is considered indicative of a good fit. For other goodness of fit indices, the values indicative of good fit are RMSEA <0.10 and CFI, NNFI, TLI, and IFI >0.90 (Brown and Moore, 2012; Geldhof et al., 2014).

### Known-group discriminant validity

The known-group discriminant validity was evaluated by testing for differences in the HP-CSS total score in relation to known groups of demographic characteristics (Gregory, 2012). An independent samples *t*-test and a one-way analysis of variance were performed to compare the HP-CSS total score between the different groups.

### Reliability

Cronbach's α coefficient was used to measure the internal consistency of the HP-CSS (Posner et al., 2011; Tavakol and Dennick, 2011). The odd-even split method was used to measure the split-half reliability; the HP-CSS items were divided into two parts, and the Spearman-Brown coefficients of odd-even items were calculated (Pronk et al., 2022).

## Results

### Demographic characteristics of participants

The sample includes 836 healthcare professionals. Participants' ages ranged from 18 to 29, 30 to 39, 40 to 49, and over 50 years, with percentages of 45.7, 38.2, 13.5, and 2.6%, respectively. The other data are shown in Table 1.

**Table 1:** Demographic characteristics of participants (*n* = 836).

**Variables**	**Number**	**Percentage (%)**
**Age (years)**		
18–29	382	45.7
30–39	319	38.2
40–49	113	13.5
≥50	22	2.6
**Gender**		
Men	33	3.9
Women	803	96.1
**Marital status**		
Single	290	34.7
Married	534	63.9
Spinsterhood	11	1.3
Divorced	1	0.1
**Education level**		
Vocational school of health	5	0.6
Associate degree	201	24.1
Undergraduate	581	69.5
Postgraduate	38	4.5
PhD candidate	11	1.3
**Occupation**		
Doctor	35	4.2
Nurse	767	91.7
Therapist	34	4.1
**Professional title**		
Junior	486	58.2
Intermediate	266	31.8
Deputy	77	9.2
Chief	7	0.8
**Work years as a health professional (years)**		
0–5	323	38.6
6–10	219	26.2
11–15	116	13.9
16–20	77	9.2
≥21	101	12.1
**Communication skill training**		
Yes	558	66.7
No	278	33.3

### Item analysis

The critical ratio (CR) of 18 items was >3 (CR ranging from 9.937 to 28.816), indicating the discrimination of each item was good. The scores of each item were positively correlated with the total score (*r* ranging from 0.357 to 0.778, *P* < 0.001), which showed that each item was moderately to strongly correlated with the scale (Table 2).

**Table 2:** Items analysis of HP-CSS.

**Items**	**(Mean** ±**SD)**		**Critical ratio**	**Item-total correlation**
	**Low score group (*****n*** = **251)**	**High score group (*****n*** = **244)**		
Item 1	4.90 ± 0.76	5.97 ± 0.17	−21.780^**^	0.690^**^
Item 2	4.69 ± 0.84	5.89 ± 0.36	−20.875^**^	0.694^**^
Item 3	4.76 ± 0.86	5.89 ± 0.44	−18.365^**^	0.659^**^
Item 4	4.77 ± 0.74	5.96 ± 0.19	−24.572^**^	0.726^**^
Item 5	4.82 ± 0.73	5.96 ± 0.22	−23.502^**^	0.719^**^
Item 6	4.37 ± 0.99	5.85 ± 0.53	−20.764^**^	0.715^**^
Item 7	4.63 ± 0.72	5.92 ± 0.27	−26.641^**^	0.756^**^
Item 8	4.33 ± 0.95	5.79 ± 0.50	−21.566^**^	0.696^**^
Item 9	4.87 ± 0.62	5.97 ± 0.18	−26.876^**^	0.734^**^
Item 10	4.09 ± 0.99	5.14 ± 1.32	−9.937^**^	0.439^**^
Item 11	4.49 ± 0.76	5.90 ± 0.32	−27.233^**^	0.773^**^
Item 12	4.64 ± 0.68	5.95 ± 0.23	−28.816^**^	0.778^**^
Item 13	4.54 ± 0.72	5.89 ± 0.32	−27.267^**^	0.746^**^
Item 14	4.92 ± 0.70	5.97 ± 0.17	−23.126^**^	0.695^**^
Item 15	4.88 ± 0.62	5.98 ± 0.16	−27.342^**^	0.729^**^
Item 16	3.04 ± 1.00	4.18 ± 1.49	−10.011^**^	0.357^**^
Item 17	4.32 ± 0.69	5.51 ± 0.63	−19.920^**^	0.615^**^
Item 18	3.53 ± 1.06	5.01 ± 0.95	−16.407^**^	0.514^**^

HP-CSS, Health Professional Communication Skills Scale; SD, standard deviation.

** P ≤ 0.01.

### Confirmatory factor analysis

**Figure 2:**
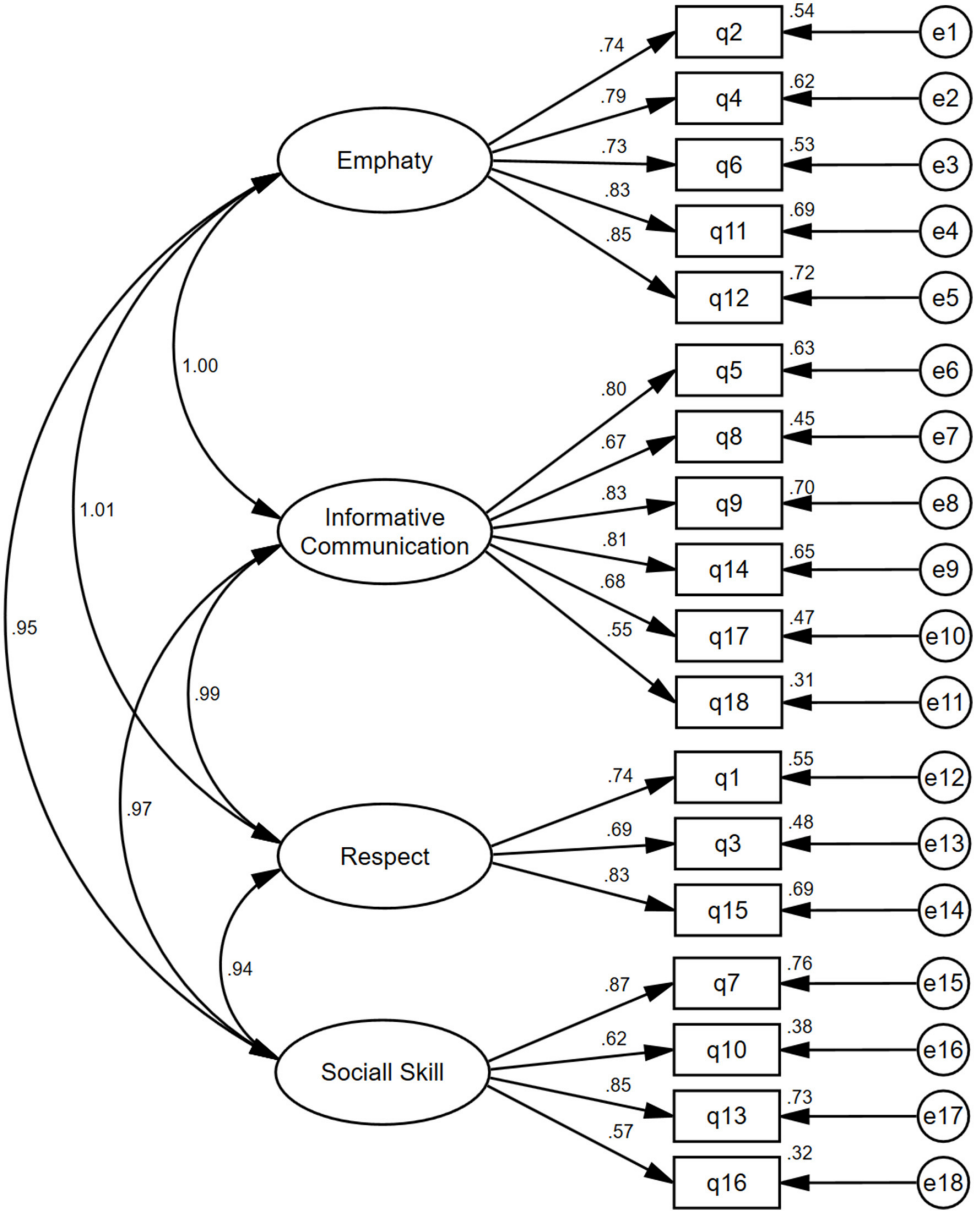
Confirmatory factor analysis of the four factor model.

The KMO value was 0.946, and the Bartlett spherical test statistic was 7,828.831 (*P* < 0.001) in the present study, demonstrating the data were suitable for factor analysis. The fit indices showed that the original correlated four-factor model of HP-CSS was adequate: χ^2^ = 722.801; df = 126; χ^2^/df = 5.737; RMSEA = 0.075; CFI = 0.923; NNFI = 0.908; TLI = 0.906; IFI = 0.923. The four-factor model is shown in Figure 2.

### Known-group discriminant validity

The HP-CSS total score was related to gender, occupation, work years, and communication skill training (*t* = −2.477, *P* = 0.013; *F* = 12.417, *P* < 0.001; *F* = 2.458, *P* = 0.044; *t* = 3.035, *P* = 0.002, respectively). The other variables were not related to the HP-CSS total score (Table 3).

**Table 3:** Differences in total score of the HP-CSS between known-groups (*n* = 836).

**Variables**	**Number**	**Total score (mean ±SD)**	**Statistics**	***P*-value**
**Age (years)**				
18–29	382	92.47 ± 9.29	1.748^b^	0.156
30–39	319	91.01 ± 10.20		
40–49	113	92.43 ± 8.70		
≥50	22	93.68 ± 8.07		
**Gender**				
Men	33	87.91 ± 10.95	−2.477^a^	0.013^*^
Women	803	92.10 ± 9.47		
**Marital status**				
Single	290	92.09 ± 9.27	1.122^b^	0.339
Married	534	91.91 ± 9.67		
Spinsterhood	11	88.18 ± 11.67		
Divorced	1	–		
**Education level**				
Vocational school of health	5	95.60 ± 8.11	0.807^b^	0.521
Associate degree	201	91.31 ± 9.74		
Undergraduate	581	92.18 ± 9.64		
Postgraduate	38	90.55 ± 7.99		
PhD candidate	11	93.91 ± 7.65		
**Occupation**				
Doctor	35	92.97 ± 8.75	12.417^b^	<0.001^**^
Nurse	767	91.64 ± 9.64		
Therapist	34	97.53 ± 6.58		
**Professional title**				
Junior	486	91.85 ± 9.68	1.693^b^	0.167
Intermediate	266	91.43 ± 9.73		
Deputy	77	93.88 ± 8.25		
Chief	7	95.71 ± 4.79		
**Work years as a health professional (years)**				
0–5	323	92.00 ± 9.34	2.458^b^	0.044^*^
6–10	219	92.27 ± 9.33		
11–15	116	89.77 ± 11.36		
16–20	77	91.66 ± 9.50		
≥21	101	93.70 ± 8.17		
**Communication skill training**				
Yes	558	92.64 ± 9.46	3.035^a^	0.002^**^
No	278	90.52 ± 9.62		

HP-CSS, Health Professional Communication Skills Scale; SD, standard deviation.

a Independent sample t-test and t.

b One-way analysis of variance, F.

* P ≤ 0.05.

** P ≤ 0.01.

### Reliability

Cronbach's α coefficient was 0.922 for the total scale and ranged from 0.521 to 0.849 for each dimension. The split-half reliability was 0.865 for the total scale and ranged from 0.394 to 0.828 for each dimension (Table 4).

**Table 4:** Cronbach's alpha coefficient and split-half reliability of HP-CSS (*n* = 836).

**Variables**	**Number of items**	**Score (mean ±SD)**	**Cronbach's alpha coefficient**	**Split-half reliability**
Empathy	5	26.43 ± 3.17	0.849	0.828
Informative communication	6	30.60 ± 3.44	0.786	0.707
Respect	3	16.36 ± 1.79	0.777	0.719
Social skill	4	18.56 ± 2.57	0.521	0.394
Total of HP-CSS	18	91.94 ± 9.56	0.922	0.865

HP-CSS, Health Professional Communication Skills Scale; SD, standard deviation.

## Discussion

The ability to communicate is a crucial requirement for effective practice (Shaw et al., 2022). The present study confirmed that the Chinese version of HP-CSS has good internal consistency and construct validity among Chinese healthcare professionals. In line with research conducted in Spain (Leal-Costa et al., 2016), the results of item analysis documented that the differentiation of the HP-CSS was good between the low-score group and the high-score group, and each item was moderately to strongly correlated with the scale.

Validity reflects the extent to which the instrument can evaluate the characteristics of the objects (Yang et al., 2021). Given that the underlying factor structure of the HP-CSS has been identified, the confirmatory factor analysis was used to test the hypothesis that a relationship between the observed variables and their underlying latent constructs exists in the current study. Regarding construct validity, the HP-CSS resulted in an acceptable four-factor model, which is consistent with previous studies (Leal-Costa et al., 2016, 2020; Mendi et al., 2020).

In terms of known-group discriminant validity, the HP-CSS total score was related to gender, occupation, years of work, and training in communication skills. Participants who were women and nurses reported higher levels of communication skills than men and doctors. These discrepancies may be explained by the influence of gender; the available body of evidence suggests that through the process of socialization and behavioral norms, women become more skilled than men at encoding and decoding emotional communication (Marchiori et al., 2008). In addition, the communication approaches of female nurses focused on the patient's emotional and psychosocial concerns and had a more egalitarian style (Curtis et al., 2013). We also found that healthcare professionals with more years of experience who had received communication skill training reported higher levels of capabilities in this respect; this finding supports previous studies that communication skills can be advanced by specific training and experience accumulation (Sanchez Exposito et al., 2018; Muddle et al., 2019; Leal-Costa et al., 2020).

Reliability is used to test the internal consistency and stability of the tool. It is generally assumed that the value above 0.7 is better, 0.6–0.699 is tolerable, 0.500–0.599 is tolerable but low, and below 0.5 is poor and better to delete (Yang et al., 2020). In our study, Cronbach's α coefficient for the total scale was above threshold values (0.70), which indicates that HP-CSS has adequate internal consistency (Tavakol and Dennick, 2011). Although Cronbach's α coefficient for the social skill dimension is lower compared with previous findings (Leal-Costa et al., 2016; Mendi et al., 2020), the value is still within tolerable limits. In terms of the split-half reliability, in addition to the social skill subscale, all values of HP-CSS were good and accepted (>0.7). However, the split-half reliability was a little low for the social skill subscale of the HP-CSS; this might be attributed to the low number of items (four items). In general, the current study proved that the HP-CSS is a robust tool to assess healthcare professional communication skills in the Chinese cultural context.

## Limitations and perspectives

There were some limitations in the current study. First, this study recruited participants from three tertiary hospitals in Chengdu, Sichuan Province. Thus, the sample can only reflect the condition of southwest China. Further studies should be made in other types of hospitals and the rest of the country in China. Second, the sample mainly consisted of nurses. Future studies should validate the Chinese version among a wider population, such as pharmacists. In addition, longitudinal studies are recommended to explore the level of healthcare professional communication skills in the future.

## Data availability statement

The original contributions presented in the study are included in the article/supplementary material, further inquiries can be directed to the corresponding authors.

## Ethics statement

The Ethics Committee approval of the study was given by the West China Second University Hospital, Sichuan University before conducting the study. Additionally, verbal consent was obtained from each of the participants.

## Author contributions

XZ and FT: contributed to conception and design, investigation, data analysis and interpretation, and drafted manuscript. XG: contributed to conception and design, data curation, methodology, and critically revised manuscript. DLa, YL, and SL: contributed to investigation and data curation. XY: contributed to investigation and critically revised manuscript. DLi: contributed to critically revised manuscript. CL-C: contributed to conceptualization and critically revised manuscript. All authors contributed to the article and approved the submitted version.
